# Virological Response in Cerebrospinal Fluid to Antiretroviral Therapy in a Large Italian Cohort of HIV-Infected Patients with Neurological Disorders

**DOI:** 10.1155/2012/708456

**Published:** 2012-08-26

**Authors:** Maria Letizia Giancola, Patrizia Lorenzini, Antonella Cingolani, Francesco Baldini, Simona Bossolasco, Teresa Bini, Laura Monno, Giovanna Picchi, Antonella d'Arminio Monforte, Paola Cinque, Valerio Tozzi, Andrea Antinori

**Affiliations:** ^1^Clinical Department, National Institute for Infectious Diseases “Lazzaro Spallanzani” IRCCS, Via Portuense, 292, 00149 Rome, Italy; ^2^Institute of Infectious Diseases, Catholic University, 00168 Rome, Italy; ^3^Department of Infectious Diseases, San Raffaele Scientific Institute, 20127 Milan, Italy; ^4^Clinic of Infectious Diseases, San Paolo Hospital, University of Milan, 20142 Milan, Italy; ^5^Clinic of Infectious Diseases, University of Bari, 70124 Bari, Italy

## Abstract

The aim of the present study was to analyse the effect of antiretroviral (ARV) therapy and single antiretroviral drugs on cerebrospinal fluid (CSF) HIV-RNA burden in HIV-infected patients affected by neurological disorders enrolled in a multicentric Italian cohort. ARVs were considered “neuroactive” from literature reports. Three hundred sixty-three HIV-positive patients with available data from paired plasma and CSF samples, were selected. One hundred twenty patients (33.1%) were taking ARVs at diagnosis of neurological disorder. Mean CSF HIV-RNA was significantly higher in naïve than in experienced patients, and in patients not taking ARV than in those on ARV. A linear correlation between CSF HIV-RNA levels and number of neuroactive drugs included in the regimen was also found (*r* = −0.44, *P* < 0.001). Low -plasma HIV-RNA and the lack of neurocognitive impairment resulted in independently associated to undetectable HIV-RNA. Taking nevirapine or efavirenz, or regimen including NNRTI, NNRTI plus PI or boosted PI, was independently associated to an increased probability to have undetectable HIV-RNA in CSF. The inclusion of two or three neuroactive drugs in the ARV regimen was independently associated to undetectable viral load in CSF. Our data could be helpful in identifying ARV regimens able to better control HIV replication in the CNS sanctuary, and could be a historical reference for further analyses.

## 1. Introduction

 One of the major concerns about antiretroviral (ARV) therapy is the question of whether current ARV regimens are effective in suppressing HIV-1 replication in the central nervous system (CNS) as well as in plasma. CNS is considered one of the anatomic reservoirs of HIV replication, sites in which the cellular HIV replication has a longer half-life [1, 2]. HIV dynamics in CNS and plasma can evolve independently, leading to virologic compartmentalization of HIV infection in the CNS [3]. It is well known that HIV can evolve and replicate in neurological compartment independently from plasma and the virological response in these two different compartments can be quite different [3–5]. Moreover, a residual HIV replication in CNS and persistent intrathecal immune activation can be detected also in patients on ARV [6, 7].

To assess the replication of HIV in CNS is not easy. The levels of HIV-1 RNA in cerebrospinal fluid (CSF) has been considered an indirect measure to assess active infection in brain tissue and a biological marker of HIV infection, as well as in plasma [8]. The diagnostic and prognostic role of the detection of HIV-1 RNA in CSF for the development of neuropsychological impairment has been evidenced in HIV-infected patients [9–11]. However, in the highly active antiretroviral therapy (HAART) era the relationship between CSF HIV-RNA levels and neurocognitive impairment seems to be lost [12] and biological markers of brain damage are lacking.

The strong beneficial effect of the potent antiretroviral regimens on disease progression is clearly documented [13, 14], but the effect on the CNS and the protective role against neurologic complication of HIV infection is less evident. In the last years, a marked decline of neurologic complications has been observed [15, 16]. A decrease in the incidence of HIV-associated neurocognitive impairment has been also registered, while its prevalence has risen [17]. Cumulating evidences indicate that a relevant proportion of HIV-infected patients continue to present neurocognitive impairment despite the treatment with HAART and that currently available ARV regimens are often inadequate to treat HIV-associated neurocognitive impairment [18].

HAART is demonstrated to effectively reduce HIV-1 RNA levels in CSF [19, 20], but the neuroactive effect of antiretroviral drugs and the protective role of different drug classes in patients treated with HAART has to be conclusively defined.

The aim of the present study was to analyse the effect of antiretroviral therapy and single antiretroviral drugs on CSF HIV-1 RNA burden in a large cohort study group of HIV-infected patients affected by neurological disorders and to identify factors related to undetectable levels of CSF HIV-1 RNA in such cohort.

## 2. Methods

To analyse the effect of antiretroviral drugs, drug classes, and number of CNS-penetrating drugs on HIV-RNA load in CSF, a large group of HIV-infected patients affected by neurological disorders enrolled in the Italian Registry Investigative NeuroAIDS (IRINA) was studied. IRINA is a longitudinal, multicentric cohort study carried out in 45 Italian centres of infectious diseases, that since 2000 enrols HIV-infected patients affected by neurological disorders. In particular, the registry collects demographic and epidemiologic variables, natural history of HIV infection, antiretroviral therapy, clinical and radiological features, diagnostic criteria for neurological diagnosis, and virological and immunological parameters. Patients with paired CSF and plasma data available were included in the present study and were considered for the analysis. HIV-RNA levels in plasma and CSF were quantified by branched-DNA (Bayer, detection limit of 50 copies/mL, 1.69 log_10_), RT-PCR (Amplicor Roche Diagnostics, detection limit 50 copies/mL) or nucleic acid sequence-based amplification (NASBA) (Nuclisens HIV-1 QT assay Organon Teknika, detection limit of 80 (1.90 log_10_) copies/mL), depending on the assay used by each center. To account for the difference between NASBA and RT-PCR in HIV RNA quantification, values of HIV RNA by NASBA assay were divided by two. For the analysis, all HIV-RNA levels were transformed into log_10_ values. For the statistical analysis, CSF HIV-RNA were considered “undetectable” if the viral load was below the detection limit of the tool used.

The statistical analysis was performed including patients taking the drugs for which we have a larger case number of plasma-CSF paired samples.

Antiretrovirals known to have high level of penetration in CSF or to effectively suppress HIV-RNA in CSF from literature reports, were considered “neuroactive drugs.” Among the antiretrovirals prescribed to the study patients, the neuroactive drugs included: zidovudine, stavudine, lamivudine, abacavir, nevirapine, efavirenz, indinavir, lopinavir [21–30]. Lopinavir was always administered associated to a boost of ritonavir at recommended doses. Since indinavir was administered with or without the boost of ritonavir, boosted indinavir was considered as a different regimen from unboosted one.

Logistic regression was used to determine predictive factors of undetectable CSF viral load. Multivariable analysis was performed fitting three different models including variables related to antiretroviral therapy: in the first model the effect of each single drug included in the antiretroviral regimen was analyzed; in the second model the effect of different drug regimens was analyzed using the following categorization criteria: unboosted Protease Inhibitors PIs-, boosted PIs-, Non-Nucleoside-reverse-trascriptase-inhibitors- (NNRTIs-), NNRTIs-plus-PIs-, only-nucleoside-reverse-trascriptase-inhibitors- (NRTIs-) based regimens, or no therapy; in the third model the effect of the number of neuroactive drugs, as defined above, was analyzed. The Student *t*-test was employed to compare values of CSF HIV-RNA in different groups of patients (naïve-experienced, on HAART-no HAART). Correlation between log_10_ CSF HIV-RNA and number of neuroactive drugs was calculated using Pearson correlation coefficient *r*. All statistical analyses were performed by SPSS (version 11.0.1) for Windows (SPSS, Chicago, Illinois, USA). *P* values <0.05 were considered statistically significant.

## 3. Results and Discussion

### 3.1. Results

Three hundred sixty-three HIV-positive patients affected by neurological disorders and enrolled in IRINA Study, with available data from paired plasma and CSF samples, were selected for the present analysis. General characteristics of the patients included were reported in Table 1.

Median CD4 count, plasma, and CSF HIV-1 RNA were 71 cell × 10^9^/L (IQR: 22–162), 4.98  log_10_c/mL (3.81–5.44) and 3.63  log_10_c/mL (2.17–4.83), respectively. In 16.5% of patients CSF HIV-RNA was undetectable. Neurologic disorders included HIV encephalopathy (28.4%), Progressive Multifocal Leucoenkephalopathy (15.4%); encephalopathies of unknown origin (10.2%); Toxoplasmic encephalitis (9.9%); cryptococcosis (9.6%); cerebral lymphoma (5%); Tuberculous meningitis (2.8%); other diseases (18.7%).

Regarding antiretroviral (ARV) therapy exposure, 182 (50.1%) patients were ARV experienced and 120 (33.1%) were taking ARV therapy at diagnosis of opportunistic or neoplastic neurological disorder. The frequency of each ARV agent included in the HAART regimen were as follows: zidovudine 47 patients (12.9%), didanosine 23 patients (6.3%), stavudine 60 patients (16.5%), lamivudine 93 patients (25.6%), abacavir 16 patients (4.4%), nevirapine 14 (3.9%), 26 efavirenz patients (7.2%), indinavir 15 patients (4.1%), ritonavir-boosted indinavir 8 patients (2.2%), nelfinavir 25 patients (6.9%), ritonavir-boosted lopinavir 17 patients (4.7%). Regarding drug regimens, 37 (10.2%) patients were taking unboosted PIs-, 30 (8.3%) boosted PIs-, 31 (8.5%) NNRTIs-, 8 (2.2%) NNRTIs plus PIs-, and 14 (3.9%) NRTIs-based regimens.

Eight patients (2.2%) were taking one neuroactive drug, as above defined, 45 (12.4%) were taking two neuroactive drugs, and 67 (18.5%) were taking three or four neuroactive drugs.

**Figure 1:**
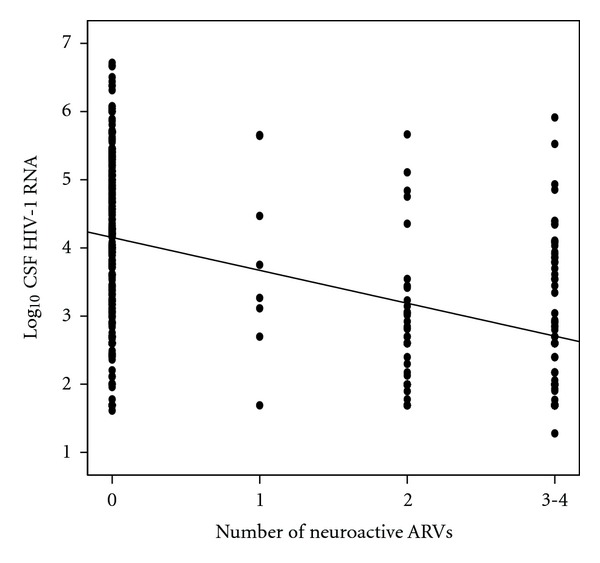
Correlation between the HIV-RNA load in the cerebrospinal fluid (CSF) (copies/mL) and the number of neuroactive drugs included in the HAART regimen. *r* = −0.44, *P* < 0.001. ARVs: antiretroviral drugs.

Mean CSF HIV-1 RNA was significantly higher in naïve (4.3 (SD: ±1.3) log_10_c/mL) than in experienced (3.2 (±1.2) log_10_c/mL) patients (*P* < 0.001, Student *t*-test). Similarly, mean CSF HIV-1 RNA was significantly higher in patients not taking ARV therapy (4.2 (±1.2) log_10_c/mL) than in patients on ARV therapy (2.9 (±1.1) log_10_c/mL) (*P* < 0.001, *t*-Student test). A linear correlation between the CSF HIV-1 RNA levels and the number of neuroactive drugs included in the HAART regimen was also found (*r* = −0.44, *P* < 0.001) (Figure 1). Furthermore, analyzing the effectiveness of antiretrovirals included in the patients' regimens using the penetration score proposed by Letendre et al. [31], the significant correlation between HIV-1 RNA load in CSF and the CNS penetration-effectiveness score was confirmed (*r* = −0.43, *P* < 0.001).

Low plasma HIV-RNA and the absence of neurocognitive impairment resulted in independently associated to undetectable HIV-RNA levels in all the three models of analysis employed (Table 2). A significant correlation between HIV-1 RNA load and the evidence of neurocognitive impairment was also found (*r* = 0.11, *P* < 0.041). Regarding ARV drugs, taking nevirapine (OR: 4.46; 95% CI: 1.03–19.32, *P* = 0.045) or efavirenz (OR: 4.87; 95% CI: 1.16–20.54, *P* = 0.031) was independently associated to an increased probability to have undetectable HIV-RNA levels in CSF. Regarding ARV regimens, the use of a regimen including NNRTI (12.46 (3.28–47.41), *P* < 0.01), NNRTI plus PI (10.42 (1.59–68.46), *P* = 0.015) or boosted PI (5.64 (1.31–24.25), *P* = 0.02) was independently associated to an increased probability to have undetectable HIV-RNA levels in CSF. Similarly, the inclusion of two or three neuroactive drugs in the ARV regimen was independently associated to undetectable viral load in CSF (for two neuroactive drugs the adjusted OR was 4.11 (95% CI: 1.22–13.79), *P* = 0.022, for three neuroactive drugs the adjusted OR was 5.48 (1.94–15.48, *P* = 0.001)). Furthermore, using the CNS penetration-effectiveness rank proposed by Letendre et al. [31] was associated to a significant probability to obtain undetectable HIV-1 RNA in CSF (OR 1.20 (per 1 score higher, 95% CI 1.07–1.35, *P* = 0.001)).

No effect of neurologic disorders and of baseline CD4 cell count on HIV control in plasma and CSF was observed.

Considering the subgroup of 120 patients taking HAART at neurological diagnosis and antiretroviral classes (PI, boosted PI, NNRTI, NNRTI plus PI, only NRTI) as cofactor, NNRTI-containing regimen was the only predictive factor of CSF undetectability (OR: 5.38; 95% CI: 1.52–19.00, PI-regimen as reference).

### 3.2. Discussion

The goal of the long lasting therapeutic strategy in HIV-infected patients must consider the complete control of HIV replication not only in the periphery, but also in the neurological compartment. This is especially true for patients with neurological complications affecting the CNS. A lot of reasons can partially explain the particular condition of CNS compartment: first of all, the presence of the blood brain barrier (BBB) in the CNS, with the tight junctions between the endothelial cells that make peculiar the CNS from the point of an anatomic view, separating the brain from the rest of the body. The CNS can be considered a sanctuary of HIV infection, where drugs penetrate in variable proportion. Some antiretrovirals penetrate less effectively, reaching sometimes inadequate concentrations. Drug penetration is based on different conditions: molecular weight, lipid solubility and protein binding for diffusion, active transport system, and drug efflux system. In presence of low concentrations of drugs in CSF the replication of HIV can continue. Some drugs are considered to have good penetration in cerebral compartment and to have efficacy on controlling HIV replication [21–30].

The issue if the use of drugs having a good penetration across the BBB is necessary to reach the control of HIV replication also in CNS is currently not clear. It is also questioned if a complete suppression of HIV viral load can be reached in CSF and if it is possible to identify an optimal antiretroviral therapy to obtain the complete control of HIV replication in CSF.

The use of nucleotide analogues has been associated to AIDS dementia decline in EUROSIDA cohort [15], but only for zidovudine a controlled trial has demonstrated a beneficial effect on dementia complex [32].

Previous studies showed a better virological decline of HIV-RNA in CSF using three or more drugs with good penetration [19] and a higher number of CSF-penetrating drugs [33–35]. The use of HAART was correlated to the decline of HIV-RNA in CSF and to a better neurocognitive performance [34, 36] in some patients, but the use of single CSF penetrating HAART versus multiple has not shown a marked benefit in psychomotor speed change in nonadvanced patients [37]. In a previous study, we failed to find a correlation between the neurocognitive performance (NPZ8 score) and the number of penetrating drugs included in the antiretroviral regimen in HIV-positive patients with a good immunological level and stable HAART [38].

A penetration score has been proposed by Letendre et al. [31] to evaluate whether the penetration of antiretrovirals in the CNS is associated to lower CSF viral load. A numeric penetration score was obtained summing the score assigned to singular drugs included in the antiretroviral regimen taken by patients, considering the published data on CSF concentrations and chemical properties. Higher penetration scores were strongly and independently associated with lower CSF viral load also after adjusting for total number of antiretrovirals and plasma viral load [31].

The data obtained from the present study, conducted on a large cohort of HIV-infected patients, confirm that antiretroviral therapy can determine a significant reduction of HIV burden in CSF as documented by the lower HIV-1 RNA load observed in antiretroviral experienced patients compared to naïve patients, and in HAART-treated patients compared to non-HAART-treated patients, also in presence of neurological disorders. Our data indicate that a higher number of CNS penetrating ARVs or an higher CNS penetration-effectiveness score using Letendre classification of ARVs correlated with lower CSF RNA levels (*r* = −0.44, *P* < 0.001, *r* = −0.43, *P* < 0.001, resp.). Moreover, the use of a higher number of CNS-penetrating drugs enhances the probability to obtain undetectable level of CSF HIV-RNA. Compared to regimens containing no CNS-penetrating ARVs, the use of two (OR = 4.11; 95% CI = 1.22–13.79) or at least three (OR = 5.48; 95% CI = 1.94–15.48) penetrating CNS ARVs markedly improved the probability of having a CSF HIV RNA level below the detection limit of 50 copies/mL.

Furthermore, we made an effort to identify antiretroviral schemes or agents that could improve HIV control in CSF. Among specific ARVs, the use of nevirapine (OR = 4.46; 95% CI = 1.03–19.32) and efavirenz (OR = 4.87; 95% CI = 1.16–20.54) showed the best correlations with the probability of having CSF HIV RNA level below the detection limit of 50 copies/mL. Among antiretroviral drug classes, the exposure to NNRTIs (OR = 12.46; 95% CI = 3.28–47.4) and boostered PI (OR = 5.64, 95% CI = 1.31–24.25) increases the probability to reach undetectable levels of HIV-RNA in CSF. Taken together these data indicate that the use of ARVs with good penetration into the CNS increases the probability of controlling HIV replication in CSF. Prospective study are needed to confirm our data.

We are aware of potential limits of our study. First, we know that the undetectable HIV load in CSF cannot fully reflect controlled replication of HIV in the brain tissue. Moreover, we have no data to support the hypotheses that controlled HIV replication in CSF translates in neurocognitive improvement in our study patients on HAART.

In recent years, a decreased frequency of HIV-related neurological disorders was observed, and a lower number of patients underwent lumbar puncture, so the number of available paired CSF-plasma samples was lower. The analysis here reported was limited to drugs for which we have a larger case number of plasma-CSF paired samples. It does not include new NRTIs, NNRTIs, PIs drugs and new classes, as fusion and entry inhibitors or integrase inhibitors, more recently introduced, that are very interesting to study, and it represents a limitation of the present study. Unfortunally, because of the small number available for statistics, we were not able to investigate these more recent drugs.

In conclusion, our data support the concept that the inclusion of a higher number of CNS penetrating drugs is associated with an increased probability of having undetectable CSF HIV RNA levels in HIV-infected patients affected by neurological disorders. Our data could be helpful in identifying ARV regimens able to better control HIV replication in the CNS sanctuary and could be a historical reference for further analyses regarding the “new antiretroviral drugs”.

## Figures and Tables

**Table 1:** General characteristics of the 363 HIV-positive patients included in the study.

Characteristics	Patients = 363
Male gender (*n*, %)	281 (77.4%)
Age, median (years)	41 (IQR, 36–46)
HIV transmission route (*n*, %)	
(i) IVDU	157 (43.3%)
(ii) MSM	46 (12.7%)
(iii) Heterosexual	111 (30.5%)
(iv) Other/unknown	49 (13.5%)
Previous AIDS defining event (*n*, %)	109 (30.0%)
CD4 cell count, median (cell/mm^3^)	71 (IQR, 22–162)
Plasma HIV-1 RNA, median (log_10_cp/mL)	4.98 (IQR, 3.81–5.44)
CSF HIV-1 RNA, median (log_10_cp/mL)	3.63 (IQR, 2.17–4.83)
Undetectable CSF HIV-RNA (*n*, %)	60 (16.5%)
Experienced to antiretroviral therapy (*n*, %)	182 (50.1%)
Experienced on ARV at neurological diagnosis (*n*, %)	120 (33.1%)
Time on HAART, median (months)	16 (IQR, 5–41)
>6 months on HAART before diagnosis (*n*, %)	118 (32.5%)
Cognitive symptoms	213 (58.7%)
Abnormal mental status	90 (24.8%)
Cerebral atrophy	137 (37.7%)
Neurological disorders	
(i) HIVE	103 (28.4%)
(ii) PML	56 (15.4%)
(iii) PCNSL	18 (5.0%)
(iv) TE	36 (9.9%)
(v) EUO	37 (10.2%)
(vi) CM/TB	45 (12.4%)
(vii) Other diseases	68 (18.7%)

IVDU: intravenous drug users, MSM: men who have sex with men, CSF: cerebrospinal fluid; HIVE: HIV encephalopathy; PML: progressive multifocal leucoencephalopathy; PCNSL: primary central nervous system lymphoma; TE: toxoplasmic encephalitis; EUO: encephalopathies of unknown origin; CM: cryptococcosis; TB: CNS tuberculosis/tubercular meningitis.

**Table 2:** Factors related to undetectable HIV-RNA levels in cerebrospinal fluid (CSF) at logistic regression model adjusted for age, gender, HIV-transmission route, time on HAART before neurological diagnosis (more or less than 6 months), abnormal mental status, cerebral atrophy, and neurological disorder.

Variables	Crude OR (95% CI)	*P*-value	Model 1		Model 2		Model 3	
			Adjusted OR (95% CI)	*P*-value	Adjusted OR (95% CI)	*P*-value	Adjusted OR (95% CI)	*P*-value
Plasma HIV-RNA (log10 cp/mL)	0.57 (0.47–0.69)	<0.01	0.70 (0.51–0.95)	0.024	0.70 (0.51–0.96)	0.027	0.68 (0.51–0.91)	0.009
CD4 at diagnosis	1.05 (0.97–1.14)	0.221	0.97 (0.86–1.10)	0.680	0.96 (0.85–1.09)	0.557	0.96 (0.86–1.08)	0.473
(50 cells increase)								
Drugs at diagnosis								
(i) AZT	3.60 (1.83–7.08)	<0.01	2.15 (0.51–9.04)	0.295				
(ii) DDI	2.37 (0.93–6.04)	0.071	1.67 (0.37–7.65)	0.508				
(iii) D4T	3.65 (1.95–6.83)	<0.01	1.53 (0.41–5.65)	0.527				
(iv) 3TC	4.53 (2.54–8.10)	<0.01	1.62 (0.39–6.66)	0.505				
(v) ABV	1.17 (0.32–4.25)	0.807	0.22 (0.03–1.68)	0.144				
(vi) NVP	4.10 (1.37–12.28)	0.012	4.46 (1.03–19.32)	0.045				
(vii) EFV	7.38 (3.21–16.95)	<0.01	4.87 (1.16–20.54)	0.031				
(viii) IDV	1.90 (0.58–6.17)	0.288	0.86 (0.16–4.50)	0.856				
(ix) IDV/r	3.14 (0.73–14.49)	0.125	2.06 (0.31–13.58)	0.454				
(x) NFV	1.66 (0.63–4.35)	0.302	0.68 (0.16–2.78)	0.587				
(xi) LPV/r	2.21 (0.75–6.51)	0.152	1.10 (0.22–5.64)	0.907				
Drug classes								
(i) No ARV	1.00				1.00			
(ii) NNRTIs-based regimens	14.32 (6.13–33.44)	<0.01			12.46 (3.28–47.4)	<0.001		
(iii) PIs-based regimens	3.50 (1.54–7.96)	<0.01			2.48 (0.75–8.22)	0.138		
(iv) Boosted PIs-based regimens	6.88 (2.42–19.52)	<0.01			5.64 (1.31–24.25)	0.020		
(v) Only NRTIs	3.22 (0.83–12.53)	0.092			1.25 (0.09–17.10)	0.866		
(vi) PI + NNRTIs-based regimens	7.07 (1.57–31.89)	0.01			10.42 (1.59–68.46)	0.015		
Number of neuroactive drugs								
(i) 0	1.00						1.00	
(ii) 1	1.68 (0.20–14.41)	0.634					1.67 (0.16–17.82)	0.673
(iii) 2	6.50 (3.01–14.04)	<0.01					4.11 (1.22–13.79)	0.022
(iv) 3-4	6.58 (3.32–13.05)	<0.01					5.48 (1.94–15.48)	0.001
Cognitive symptoms	0.47 (0.27–0.83)	<0.01	0.38 (0.16–0.90)	0.029	0.38 (0.16–0.88)	0.025	0.41 (0.18–0.95)	0.038

OR: odds ratio; IVDU: intravenous drug users; MSM: men who have sex with men; AZT: zidovudine; DDI: didanosine; D4T: stavudine; 3TC: lamivudine; ABV: abacavir; NVP: nevirapine; EFV: efavirenz; IDV/r: indinavir/ritonavir; IDV: indinavir; NFV: nelfinavir; LPV/r: lopinavir/ritonavir; ARV: antiretroviral therapy; NNRTI: nonnucleoside reverse trascriptase inhibitors; PI: protease inhibitors; NRTI: nucleoside reverse trascriptase inhibitors; HAART: highly active antiretroviral therapy; HIVE: HIV encephalopathy; PML: progressive multifocal leucoencephalopathy; EUO: encephalopathies of unknown origin; TE: toxoplasmic encephalitis; PCNSL: primary central nervous system cerebral lymphoma; CM: cryptococcosis; TB: CNS tuberculosis/tubercular meningitis.
